# Embedded Computation Architectures for Autonomy in Unmanned Aircraft Systems (UAS)

**DOI:** 10.3390/s21041115

**Published:** 2021-02-05

**Authors:** Luis Mejias, Jean-Philippe Diguet, Catherine Dezan, Duncan Campbell, Jonathan Kok, Gilles Coppin

**Affiliations:** 1 Queensland University of Technology, Brisbane, QLD 4000, Australia; 2 CROSSING, CNRS, Adelaide, SA 5000, Australia; jean-philippe.diguet@cnrs.fr; 3 Lab-STICC, UBO University, 29238 Brest, France; Catherine.Dezan@univ-brest.fr; 4 University of South Australia, Adelaide, SA 5000, Australia; da.campbell@outlook.com.au; 5 Australian Institute of Marine Science, Townsville MC, QLD 4810, Australia; j.kok@aims.gov.au; 6 Lab-STICC, IMT Atlantique School, 29238 Brest CEDEX 03, France; gilles.coppin@imt-atlantique.fr

**Keywords:** UAS, autonomy, computing architectures, UAS applications

## Abstract

This paper addresses the challenge of embedded computing resources required by future autonomous Unmanned Aircraft Systems (UAS). Based on an analysis of the required onboard functions that will lead to higher levels of autonomy, we look at most common UAS tasks to first propose a classification of UAS tasks considering categories such as flight, navigation, safety, mission and executing entities such as human, offline machine, embedded system. We then analyse how a given combination of tasks can lead to higher levels of autonomy by defining an autonomy level. We link UAS applications, the tasks required by those applications, the autonomy level and the implications on computing resources to achieve that autonomy level. We provide insights on how to define a given autonomy level for a given application based on a number of tasks. Our study relies on the state-of-the-art hardware and software implementations of the most common tasks currently used by UAS, also expected tasks according to the nature of their future missions. We conclude that current computing architectures are unlikely to meet the autonomy requirements of future UAS. Our proposed approach is based on dynamically reconfigurable hardware that offers benefits in computational performance and energy usage. We believe that UAS designers must now consider the embedded system as a masterpiece of the system.

## 1. Introduction

Unmanned Aircraft Systems (UAS), commonly referred to as drones, are reported to be the breakthrough and transformative technology that will provide huge economic benefits globally [1]. The Teal Group alone forecasts global UAS production to reach USD$93 billion within the next 10 years, with 28% of this value residing in the civilian domain. The greatest areas of economic growth benefiting from the application of UAS technology are agriculture, asset management, mining and construction sectors. The list of application areas is rapidly expanding, but includes disaster response, search and rescue, wildlife monitoring, real estate photography, media and many more. With the growing use of UAS across many sectors of society and industry comes a series of requirements and specifications that needs to be met before this occurs. Whereas the platform technology is developing rapidly and affordability increasing, there remains the issue of upholding acceptable safety standards and airspace integration with other airspace users including manned aviation.

The UAS that are more likely to be used in the application areas indicated above are small or very small, depending on the definitions adopted by national regulatory authorities. These platforms will have greater limitations in size, weight and power (electrical) (SWaP) compared to their larger counterparts. From a technology capability perspective, current UAS can perform multiple tasks such as collision avoidance, SLAM (simultaneous localization and mapping) navigation, waypoint navigation, autonomous takeoff and landing, path planning and decision making, among others, simultaneously. If we then consider the emerging drivers of beyond visual line-of-sight (BVLOS) operations, airspace integration, functional safety, autonomy, multiple UAS operations, industrial internet of things and cognitive functions, the computation demands coupled with SWaP constraints of the onboard embedded computers is drawn into question. This computational demand comes with the cost of requiring high speed and powerful embedded computers that more often than not require high power. This scenario might be achievable in large UAS (such military UAS) in which size, weight and power (SWaP) are not limiting factors. Unfortunately, these types of UAS are not likely to be cost effective for civilian applications.

Mini or micro UAS are a beneficial technology in civilian and military contexts due to attributes such as easy transport, low power consumption, rapid deployment and configurable payloads. These UAS are generally easy to deploy in remote areas at much reduced costs. However, they lack the ability to carry heavy payloads, namely computers, batteries and sensors. In this scenario, a new onboard computing paradigm needs to be proposed to address these limitations.

Embedded, low power and reconfigurable hardware offers a feasible alternative to reduce the burden of carrying payloads without compromising computing capability. Reconfigurable computing has become an alternative to performing multiple onboard tasks that are potentially computing intensive at no extra cost in terms of SWaP.

Furthermore, UAS in civilian contexts need to deal with the inherent unpredictability and dynamic nature of real world environments. This requires the design of new computing architectures that are flexible and fast enough to respond to environments in which information might not be accurate or certain. In these cases, UAS must be enabled with levels of autonomy, cognition and reasoning that will demand computing resources from the onboard systems.

In this paper, we provide an analysis of what autonomy means in terms of processing tasks and resulting embedded computing requirements. We analyse the most common tasks onboard UAS and their relationship with the applications. Our methodology is based on three stages. At first, and detailed in Section 2, we draw a relationship a between tasks and autonomy levels. Then we study and quantify, considering an emerging case study and perspectives, the impact a UAS AL (Autonomy Level) has on the computation platform in Section 5. Finally, in Section 6, we analyse the computing demand and draw conclusions about a possible promising architecture and associated design methodology.

## 2. Relating UAS Applications and Autonomy

In this section, we present an overview of the most common tasks executed onboard an unmanned aircraft and attempt to define the relationship between these tasks and the level of autonomy. We argue that the higher in the autonomy definition a UAS is required to operate, an increase in the number of tasks running onboard will be expected when compared to lower autonomy levels. Hence, increasing the autonomy level will then impose demands on the computational resources needed.

### 2.1. Existing Attempts to Define Autonomy in Autonomous Systems

The International Civil Aviation Organization (ICAO) [2] classifies unmanned aircraft into two categories under the Circular 328 AN/190 [3]: remotely piloted aircraft and autonomous aircraft. In this work, we use the term *UAS* with reference to *autonomous aircraft*, noting that at times a piloted takeoff/landing might be in place while the rest of the mission is autonomous. UAS typically consists of Unmanned Aerial Vehicle(s) (UAV), also referred to as unmanned aircraft, ground control station(s), control link and other related support equipment working harmoniously to conduct its mission successfully. UAS in this sense, have not yet reached their full potential in civilian settings. Herein, *autonomy* is defined as proposed by the National Institute for Standards and Technology [4]:

“An unmanned system’s own ability of integrated sensing, perceiving, analyzing, communicating, planning, decision-making, and acting/executing, to achieve its goals as assigned by its human operator(s) through designed Human-Robot Interface or by another system that the unmanned system communicates with.”

Attempts to define levels of autonomy are not new. Dating back to the 1970s, one the most used classification definitions was by Sheridan [5]. In this work, ten levels of autonomy ranging from human in full control to computer in full control are presented. A revised version was later presented in [6]. This early definition has constituted the foundation to many modern autonomy classifications [7]. Nowadays, most autonomy classifications are based on the Observe-Orient-Decide-Act (OODA) framework proposed by the US Air Force [8]. For autonomous systems, the US Air Force has used this framework to define 11 levels of autonomy [8,9]. The ALFUS framework [10] is another common classification tool to define autonomy levels. Recently, an extension of this framework was presented by Kendoul [11]. Organisations such as NATO have also proposed frameworks that define human level of interaction with automation, a well known framework is the Policy for (pilot) Authorisation and Control of Tasks (PACT) [12]. Other generic attempts to define automation levels for UAS include [13,14,15,16].

**Figure 1:**
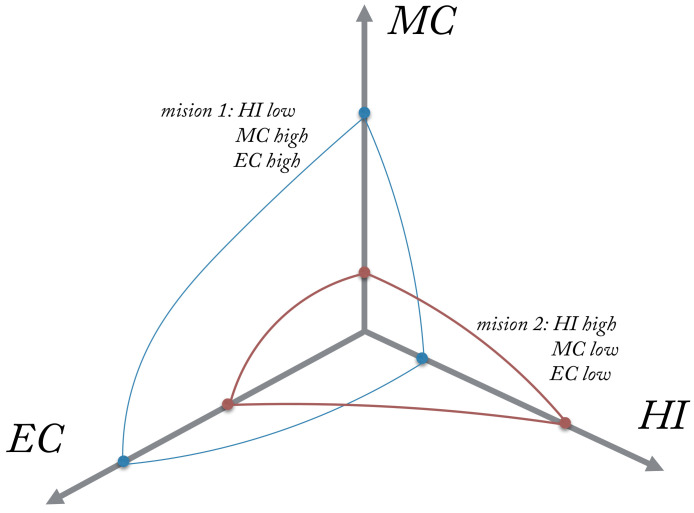
Autonomy model in the ALFUS framework. Two hypothetical missions are shown to exemplify the relationship between mission complexity (*MC*), environmental complexity (*EC*) and human independence (*HI*).

In this paper, to capture the performance of a UAS from the technical and operational perspectives, the ALFUS performance model will be used [17]. In the ALFUS framework, an autonomy level is defined by weighting a metric score for three aspects namely human independence (HI), mission complexity (MC), and environmental complexity (EC). This framework, visually, places each of the three aspects in an axis, and then determines the overall autonomy of the UAS with the required level of *human independence* to perform under a given *mission complexity*, while remaining in safety boundaries related to *environment complexity* (Figure 1).

Some of the criteria (see [17] for a complete list) used to assess the degree of complexity or difficulty for each axis are for example:

- *HI*: UAS degree of sensing environmental phenomena, UAS degree of understanding and analysing perceived situations, what/when a larger portion of the mission plan is generated by the UAS, UAS ability to generate high-level, complex plans as opposed to low-level, straightforward plans, degree of communication with the UAS and number of decisions per unit of time.
- *MC*: Mission time constraint, precision constraints and repeatability in navigation, manipulation, detection, perception, level of collaboration required, concurrence and synchronization of events and behaviours, resource management and ammunition and authority hierarchy for data access and plan execution.
- *EC*: Electromagnetic interference, use of absolute and fiducial reference points to facilitate navigation and reduce the complexity, objects size, type, density and intent; including natural or man made, lighting conditions and man-made structures.

### 2.2. UAS Applications and Autonomy

**Figure 2:**
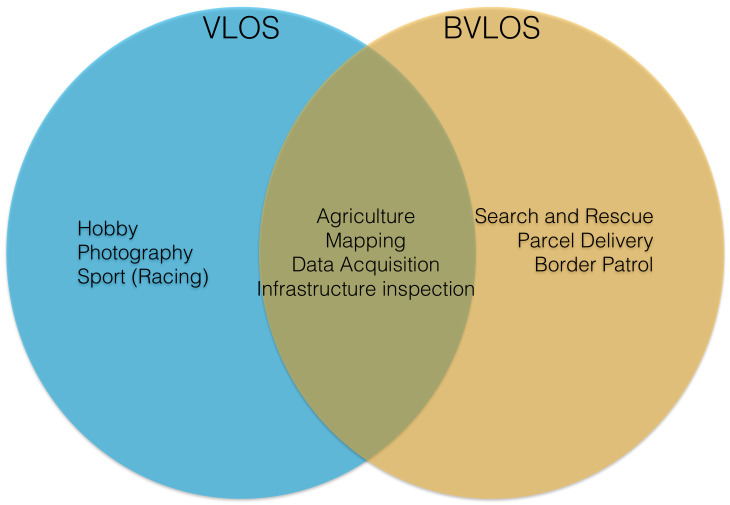
Visual line-of-sight (VLOS) and beyond VLOS (BLOS) categorisation for several common Unmanned Aircraft System (UAS) applications. Some of these application can be considered in the intersection of the two categories, i.e., can be performed by either VLOS or BVLOS.

Without doubt, the definition of autonomy level has to account for many factors, e.g., multidimensional. In this paper, of particular interest are mission complexity (MC) and environmental complexity (EC) because they establish the degree of human independence (HI) or in other words, the level of autonomy. Human independence is used in this paper as the degree in which a UAS relies on humans to operate and make decisions. Hence, low HI is seen as a manual or remotely controlled vehicle, whereas high HI is autonomous. We argue that complex missions in complex environments achieve better performance when they are highly automated. Therefore, we are interested to link the type of mission and the environment in which it is conducted with the level of autonomy, and seamlessly with the degree of computational resources needed to achieve high levels of autonomy. In order to do that, we group applications into two main categories: visual line-of-sight (VLOS) and beyond VLOS (BVLOS) (see Figure 2). VLOS is defined as flying a unmanned aircraft in visual line-of-sight at all times, not obstructed by clouds or fog, trees, buildings or other structures. Typical applications in this domain include Photography, Drone Racing and Hobby. Precision Agriculture and local Infrastructure Inspection can also be conducted within VLOS, however there might be cases in which these applications can be conducted in BVLOS or extended VLOS (using humans other than pilot to keep visual contact with the aircraft). BVLOS is defined as flying a unmanned aircraft without the pilot having visual contact with the aircraft at all times. Instead, the pilot flies the aircraft by instruments from a Ground Control Centre (GCC). Common applications in this category include Search and Rescue, Parcel Delivery, Border Patrol and remote Infrastructure Inspection. In BVLOS missions, given that the operator is remote, a number of tasks will need to be automated to achieve the same degree of control and awareness as in VLOS missions. For instance, one of the most common application of UAS is that of aerial photography/film-making. This is a typical VLOS mission in which the pilot is in control of the aircraft at all times (low HI), requiring low levels of autonomy. However, applications such as Search and Rescue tend to require some degree of autonomy and some level of decision making (high HI) [18]. The categorisation of these tasks into VLOS or BVLOS is not arbitrary. If we look carefully at some of the most widely used regulations in the world [19,20], they impose considerable constraints on hobby and commercial uses of drones, one being VLOS operation. This means that most uses of drones will fall under VLOS. The operations under BVLOS exist, but are assessed/approved on a case-by-case basis.

Conducting UAS operations in either of these categories will have an impact on the degree of onboard autonomy. For instance, once the UAS operator has no visual contact with the aircraft (BVLOS), the human see-and-avoid function must be transferred to onboard the aircraft, that is, the aircraft must now be able to sense traffic, make decisions and avoid possible collisions [21]. High performance communication links between aircraft and operator could allow a degree of remote sense-and-avoid (collision avoidance), however ensuring fast reaction times will be extremely challenging making this mode of collision avoidance highly risky. Assuming an increased demand for autonomy in BVLOS operations, the complexity of both mission and environment are crucial in order to ensure the success of the task or application. That is, BVLOS operations in highly complex environments require high levels of autonomy to guarantee task success. However, the opposite is yet to be seen, i.e., high levels of autonomy can tackle highly complex environments and missions, mostly because the extent in which an autonomous aircraft can tackle this scenario depends on advances in robust perception and AI. For example, high precision data acquisition is an example application in which the requirement of capturing data with the highest accuracy possible imposes additional demands on onboard autonomy. Complexity of environment and mission is generally low or medium in applications such as precision agriculture, marine monitoring or linear infrastructure inspection. Capturing data during these applications requires advanced precise guidance, path planning and trajectory tracking in order to ensure accurate waypoint navigation, data synchronisation, data overlap and correct resolution, amongst others. It has been demonstrated that automation can perform better than the human pilot in such cases [22,23]. Operations within the VLOS category can take advantage of the UAS operator skills. We acknowledge that the combination reasoning, decision making, visual feedback and reaction time of humans can surpass the current state-of-the-art UAS navigation. Applications such as drone racing can afford operations in highly complex environments and conduct highly complex missions as long as the human remains in the loop. Currently, most racing drones have very little onboard automation (other than visual augmentation systems or First Person View). Similarly, aerial photography is an application in which the pilot is in control of the aircraft at all times. A degree of automation generally exist on the camera gimbal for object tracking and the autopilot for better manoeuvring. However, functions such as collision avoidance, navigation and guidance are still the responsibility of the pilot. Finally, applications such as parcel delivery (Section 5) conducted in either remote or urban areas will have generally medium to high complexity of the mission and environment, respectively. Therefore, the level of required autonomy will also be from medium to high, depending on the complexity. From the operational and cost effective point of view, this application it is unlikely to be performed in VLOS mode, therefore there is an evident requirement for moderate levels of autonomy.

**Figure 3:**
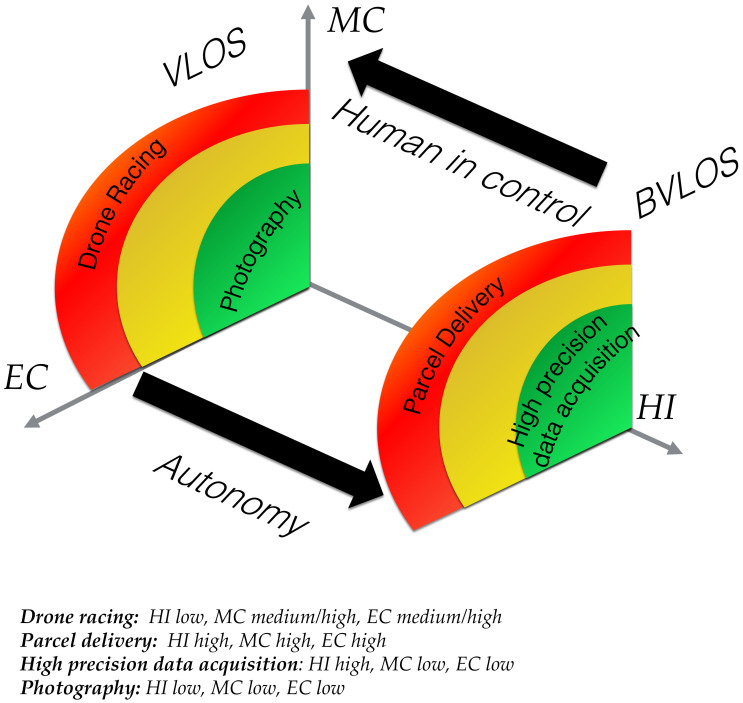
Several UAS applications can be mapped into the ALFUS model. Quantitative values can be assigned to each axis to convey an autonomy level.

Example applications referenced above can be mapped into the ALFUS model based on their requirements for EC, MC and HI. In Figure 3, we introduce four applications with their respective requirements in terms of EC, MC and HI. Qualitatively, it can be seen that Drone Racing and Photography can comfortably be placed in the MC, EC plane (e.g., VLOS) meaning they are currently performed by highly trained pilots (very low HI), whereas Data Acquisition and Parcel Delivery require moderately high levels of HI.

As previously introduced, achieving high levels of onboard autonomy requires the execution of a number of tasks concurrently [24]. The type and number of tasks are related to the final application and user preferences. However, we believe a common minimum number of tasks should be present in a highly autonomous and dependable UAS. A list of these common tasks in most UAS is presented with communication management being a high level task that has a decision making ability, whilst telemetry communication is executed at a lower level, and provides information and metrics to high level tasks. Similarly, fault detection and identification (FDI) and health management keep the same hierarchical relationship. FDI executes at a lower level providing information to other decision-making tasks executing at higher level such as health management. Refer to Section 3 for detailed descriptions of each task.

## 3. Definition and Categorisation of Onboard Unmanned Aircraft Tasks

Common to most unmanned aircraft is the separation between high and low level tasks. High level tasks are those not requiring direct access to actuators, stabilisation control loops or mission critical sensors such as accelerometers, GPS or gyros. They can be executed at high speed but do not have real-time time specifications. On the other hand, low level tasks have some real-time requirement. Direct access to actuators, state estimation filters and stabilisation control loops are common in this category. It is also common to separate the hardware each task level (high or low) is running on. For instance, embedded architectures either based on micro controllers or ARM processors such as a Cortex M7 in Pixhawk4 [25] are commonly used for low level tasks. In the case of high level tasks, it is common to find pc104 [26], mini/nano-itx/Raspberry Pis or any other small form-factor PC architecture.

A further categorisation of tasks can be made based on the function they perform onboard. Some functions could fall either in the low or high level categories previously mentioned. In this subsection, we propose a methodological classification of the most common tasks or functions an unmanned aircraft is required to execute onboard when performing a given mission. We acknowledge not all tasks are required at the same time. The intention of this list is to provide a pool of tasks that can be considered by UAS engineers when designing a UAS for a given application. We aim to provide researchers and operators with new insights to evaluate unmanned aircraft in terms of autonomy and onboard functions, and then assess the impact in the computational resources needed to achieve a given level of autonomy.

First and with similar effect to ALFUS, we classify unmanned aircraft onboard functions into five categories: (1) flight control, (2) navigation and guidance, (3) application, (4) safety and (5) mission. *Flight* pertains to low level functions implemented by the autopilot [27]. It typically implements state estimators, sensor readings, fast control and stabilisation loops, and actuator control. *Navigation and guidance* includes guidance laws and trajectory planning routines that will define and keep the aircraft on the optimal route (typically) with consideration of the application and mission priorities [23]. For instance, in this category we can find routines to plan and execute a path to flight the best route that optimises fuel, battery power consumption, data quality and time in the air. *Application* usually defines the reason the aircraft is flying. Applications such as precision agriculture [28], infrastructure inspection [29], underground inspection [30] and parcel delivery [31] are some of the uses industry is exploring with UAS. *Mission* deals with high-level tasks the UAS is responsible for beyond flying operations. It includes autonomous tasks such as mission planning [32], health monitoring [33], decision making [34] and resources management [35]. Finally, *safety* refers to the tasks a UAS must execute to ensure the safety of people and assets. It also allows the UAS to comply with regulator’s requirements to flight in civil airspace. Sense and avoid [36], airspace management [37], emergency procedures [26], fault detection and isolation [38], are common examples in this category.

### Tasks Definitions and Specifications

**Figure 4:**
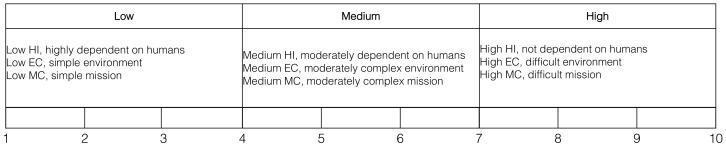
Human independence (HI), environment (EC) and mission (MC) complexities, mapped to a scale 1–10. Autonomy increases with scale.

In this section, we propose and describe some of the tasks (Table 1) that can be executed at different autonomy levels (Section 4, Figure 4). A similar list of functions for rotorcraft have been proposed by Kendoul [11]. Due to the fast paced development of UAS technology, our list represents an update to those previously proposed, categorising functions and presenting, to the best of our knowledge, the most common functions found in the literature up until now. The five categories previously introduced were flight control, navigation and guidance, application, safety and mission. The most common tasks within these categories are:

- *Flight control level:* actuator control, stabilisation and control loops, low level sensor readings, state estimation and telemetry communication.
- *Navigation and guidance level:* Static or dynamic path planning, trajectory tracking, waypoint navigation, obstacle avoidance, terrain following, vision-based navigation.
- *Application level:* Application specific sensor readings, aerial sampling, application specific camera tasks.
- *Safety level:* Sense and avoid, fault detection and identification, emergency procedures and airspace management
- *Mission level:* Energy and storage management, computational resource management, health management, decision making and communication management.

**Flight control level**

- *Actuator control:* This task could be considered as the lowest-level task on an unmanned aircraft. It involves the translation of software parameters into electrical signals for each actuator. It requires a few to tens of thousands of pulses per minute and be independent of any task that might prevent its real-time execution. Its computational load is usually negligible due the use of dedicated hardware components for each actuator.
- *State estimation and stabilisation control loops:* State estimation typically relies on a type of Kalman filter. Depending on the number of states and parameters in the aircraft dynamic model, this task could require a significant amount of computational resources. The amount of computation is mainly related to matrix operations. Floating-point computations can be very meticulous to reach accuracy and stability requirements. The control of attitude, position and speed mainly relies on close-loop control. Typical implementations involve multichannel PID approaches [39]. Due to the nature of this function (low-level), most of the computational demands are handled by using dedicated embedded hardware. However, researchers have proposed architectures where autonomy levels are linked to different layers of control [40], in which more computational resources are necessary.
- *Low level sensor readings:* This low level function accesses various digital and analog ports, such as I2C, analog to digital converters (ADCs), GPIO, RS232, PWM, USB, etc., reading and conditioning the signal before it is used by other tasks. In general terms, this function samples the ports converting sensor information into numerical values that are used by other onboard tasks. Different sample rates and scale factors are used for every sensor. In most cases, dedicated hardware is used to implement this function which minimises the requirement for computational resources. Dedicated chips handle signal level conversion, packing and unpacking of bits, clock synchronisation, device interfacing, etc.
- *Telemetry communications:* Traditionally, this task automates the process of sending data to a remote receiving equipment for monitoring. The medium can be either wired or wireless, although in UAS wireless is the most common medium using radio modems followed by 802.1xx (wifi). Nowadays, this task has evolved from simply sending data through a medium. Functions such as byte marshalling (or serialization), error checking, heart beat generation and monitoring and low level data compression are now an integral part of this task. Telemetry communications in most cases provide vital information to access whether the communication link is healthy or not, so the appropriate failsafe action is triggered.

**Navigation and guidance level**

- *Vision-based navigation:* This type of navigation have gained significant importance in recent years [41,42], primarily as a navigation method in GPS-denied environments (indoor and outdoor). Techniques derived from space localization such as visual odometry [43,44] or SLAM [45,46,47] have been tested with acceptable performances. Other techniques used in this approach are stereo vision [48,49], structure-from-motion [50,51], bio-inspired opticflow [52,53,54] and target relative navigation [55,56]. Vision-based navigation typically involves estimation of the UAS pose by computing ego-motion (camera motion). Once the aircraft state vector has been computed, control laws can be used to stabilise and guide the aircraft.
- *Path planning and trajectory tracking:* Path planning often involves finding the optimal trajectory between two points with or without consideration of obstacles. These techniques can be static (executed once) or dynamic (replanning in the event of obstacles), and involve finding the shortest path under specific constraints: (a) geometrical or kinematic constraints due to the design of the unmanned aircraft, (b) dynamics constraints defined by the environment (wind, unexpected obstacles). The trajectory tracking involves the definition of the control commands necessary to follow a set of curves/trims that respect the aerodynamic of the aircraft [57]. These curves constitute the guidance primitives for the autopilot so that it can steer the aircraft to follow a specific trajectory. Typically, these approaches rely on probabilistic, deterministic or heuristic numerical methods to compute the path while providing trajectory waypoints that already take into account the UAS constraints. Hardware implementations for discrete methods have already been investigated for deterministic and heuristic path-planning with FPGA implementation [58], but not many are investigating hardware versions of probabilistic methods [59].
- *Waypoint navigation:* This task involves the following of preplanned or manually provided waypoints (GPS coordinates). The task will generate the control commands to steer the vehicle between two consecutive waypoints. Since there is an assumption of straight line motion between two consecutive points and no strict consideration for the aircraft dynamic and kinematics constrains, this task could be considered as a simplistic version of a trajectory tracker.
- *Obstacle avoidance and terrain following:* Obstacle avoidance, as the name suggests, implies the detection and following avoidance of obstacles present in the flight path. Many passive and active sensors can be used for this purpose, and a number of algorithms have proven effective in this domain [60,61]. Terrain following compares measurements from onboard sensors with a database terrain map so the minimum clearance is respected. It can also be used for drift-free localisation purposes in case of GPS-denied environments.

**Application level**

- *Application specific sensor readings:* The distinction between low level and application specific sensor readings lies in the criticality of enabling or disabling the task. For instance, disabling accelerometers, GPS or compass readings will, in most cases, have catastrophic consequences for the UAS. On the other hand, disabling (on demand) a camera or a Lidar should not be critical for the overall navigation, unless they are used as main navigation sensor. This ability to disable a task based on the information provided by other tasks is essential in autonomous systems. For instance, stopping the Lidar or high resolution camera when onboard storage is running low, before it slows down the computer or causes a software critical failure, should be an feature in highly autonomous UAS.
- *Application specific camera tasks:* Applications that make use of cameras (video or still) are on the rise. These applications involve the recording or transmission of HD video or images that might or might not include onboard data (such as GPS, UAS orientation or altitude). Within this category, it is worth to mention several applications that have seen an increase in use such as, videography for the filming industry or sport events [62], target recognition and tracking using machine vision cameras [63], aerial photography for surveying [64], precision agriculture or real estate [65]. Depending on the configuration, each application will have an impact on the onboard processing requirements, data storage, communications and command and control links. For instance, if onboard processing is required, then computational and power resources onboard must meet the demand of applications such as target tracking [66] or video encryption [67], amongst others.
- *Aerial sampling:* Assessment of air quality is an important area of research that studies the link between poor air quality and adverse health outcomes [68]. Sampling the air close to source of pollutants may not always be possible as it can be too dangerous or risky for humans. The use of a small, lightweight unmanned aircraft can minimise the risk for humans and provide more accurate information on aerosol distribution throughout the atmospheric column. Similarly, the modality of collection and processing the samples has an impact on the computational, communications and power resources needed onboard the aircraft.

**Safety level**

- *Sense and avoid:* This task is fundamental for achieving high levels of autonomy onboard unmanned aircraft. Many of the benefits provided by UAS will come from applications that require operations beyond line-of-sight. Operating UAS in civilian airspace, which is a complex environment, is not trivial [69]. In this task we can also include a form of obstacle avoidance, either dynamic or static. Whether avoiding other aircraft or obstacles on the path, there are common functional blocks in a sense-and-avoid system that can be reused. A sense-and-avoid system can be cooperative or uncooperative, and typically encompasses sensors, detection and tracking algorithms and evasive control measures [21].
- *Fault detection and diagnosis:* This is mission critical if robust and safe UAS operations are to be conducted, and a key consideration when demonstrating dependability, safety and reliability of the UAS. Real-time techniques are preferred as they make it possible to instantly analyse onboard faults and trigger the best strategy to deal with the event. The multiple-model adaptive estimation (MMAE) approach has been applied successfully to deal with fault detection and diagnosis (FDD) problems in various flight scenarios [70,71,72]. Other approaches, have dealt with the high computational requirements of these techniques by using different estimators without loss of performance [73]. Health-management and mitigation strategies for multiple UAS have also been proposed [74].
- *Emergency procedures:* With the increased presence of unmanned aircraft flying over people and infrastructure assets, a robust and trusted system that deal with onboard emergencies is an essential capability. To intelligently and safely trigger a strategy to deal with onboard failures is one of the main challenges in manned and unmanned aviation safety. A system that can land an aircraft or adapt its flight control in response to an engine failure, actuator faults, loss of sensor readings or any other onboard failure is key in highly autonomous aircraft [26,75]. A system like this, will likely require a number of processing stages, each demanding some computational capability from onboard resources [76].
- *Airspace management:* The increasing number of unmanned aircraft flying in civilian airspace means that future airspace will be characterised by a combination of manned and unmanned aircraft. The mandatory nature of this system will be evident, because the current system for UAS authorizations is not scalable for the vast number of applications anticipated by government and industry [37,77,78]. New technologies onboard aircraft as well as ground support systems will need to be developed [13,79]. Onboard aircraft, this will mean new sensors and software tools that allows interaction and safe separation between them. This function is closely integrated with other subsystems such as guidance and navigation, decision making and collision avoidance.

**Mission level**

- *Health management:* Is the ability of a system to prevent, detect, diagnose, respond to and recover from conditions that may change the nominal operation of that system [80]. In that sense, we make the distinction between fault detection and identification (FDI) and health management, as FDI is part of the overall health management system. Health management systems are an integral part of most aircraft [81,82], however, this is a relatively novel concept in UAS. Early attempts to develop health management systems for UAS were focused on teams for persistent surveillance operations [83]. Approaches based on Bayesian networks for aircraft health-management have also been proposed in the literature [34,84]. Current challenges include efficient algorithms, embedded computing for real-time processing and simulation, validation and verification [85].
- *Communication management:* This task deals with strategies to recover or maintain the communication link between unmanned aircraft and the ground control station. It provides the ability to adjust data compression based on available channel throughput, enables data encryption when required and implements error checking for data integrity. It computes metrics to estimate the quality of the communication link, that then can be used by other subsystems for decision making.
- *Energy and storage management:* Managing energy consumption and distributing it intelligently to different subsystems based on mission goals is an essential function in any unmanned aircraft. Power consumption varies during different phases of the flight (take-off, climb, cruise and descent). It is also impacted by path planning, hence the optimisation strategies that seeks reduction in flight times and manoeuvring in most path planners. An intelligent energy management system will enable and disable subsystems based on peak power consumption, but will not manage energy consumption within these subsystems [86]. It addition, another essential system is data storage management. Nowadays, UAS can collect a considerable amount of high quality data, which imposes demands and balance between onboard processing, data transmission and data storage [87]. Managing these aspects efficiently is key in modern UAS.
- *Computational resource management:* The computational capabilities of modern CPUs and parallel processors (GPUs, FPGAs) have made possible the execution of a number of tasks concurrently. Task allocation and scheduling methods become now essential to optimally distribute computational tasks over single- or multi-core processing architectures, in order to ensure the completion of each calculation within timing requirements, without impacting the performance of other applications. Allocating fixed resources (processor time, number of cores, etc.) to each task might not be the best strategy to deal with the dynamic nature of the environment and mission goals. This dynamic nature will require flexibility to allocate the number of available cores, the amount of cache memory available and prioritise FPGA (or GPU) usage over CPU. Many factors can impact the inner decision making that allows intelligent task scheduling such as energy consumption, mission goals, aircraft internal state and requirements to handle onboard data acquisition and processing. Therefore, there is an implicit inter-task communication process between computational resource management, energy management, storage management and mission management [34].
- *Decision making:* This is arguably one of the most challenging tasks onboard unmanned aircraft. The complexity of the task is evidenced by the multiple criteria and multiple objective nature of the context. Objectives can be conflicting, therefore compromises must be made in order to achieve most critical objective(s). Each task previously mentioned will have multiple attributes that will need to be optimised in order to meet a single or multiple objectives. For instance, the path planning task will optimise attributes such as fuel, energy and time to achieve a goal(s) of reaching the destination, avoiding obstacles and/or flight under certain altitude [88,89].

## 4. Relationship between Applications and Autonomy Levels

In order to express the autonomy requirements of a given application, we map the ALFUS metrics MC, HI and EC into nine brackets on the scale 1–10 [7] (Figure 4). This scale allows us to assign quantitative values to each ALFUS metric, that can then be weighted to convey an overall UAS autonomy level (UAS AL) as a numerical value between 1–10 for a given application. This metric (UAS AL) can then be compared with existing autonomy assessment models or definitions such as ALFUS [7], RALFUS [11], Sheridan [5] or NASA [16] (Note: NASA model is scaled from 1 to 8).

With the ability to assign numerical values to each metric of the ALFUS model, we can now link applications within VLOS and BVLOS categories with the UAS AL (Figure Table 2). Successful VLOS operations owe most of their accomplishment to the pilot. Autonomy, when present, will help the pilot making his/her job effortless. For instance, automatically tracking objects using a gimbal camera. On the other hand, successful BVLOS operations, due to the lack of pilot direct control authority, owe most of the success to the degree in which pilot functions are replaced by onboard tasks, e.g, sense-and-avoid, emergency landing, obstacle avoidance, precise guidance, etc. These are functions that will increase UAS autonomy to levels in which humans will become supervisory agents. Therefore, in order to draw a relationship between a given application and the number of pilot functions that needs to be automated onboard, we have compiled several common UAS applications in Table 2.

In this table, we show the link between the number of concurrent onboard tasks that are required, in either VLOS or BVLOS operations, in order to ensure application success. We also draw the relationship between applications, the ALFUS model and UAS AL. For instance, the reasoning behind Table 2 is as follows:

- *Infrastructure Inspection* can be conducted in two modalities, local (VLOS) or remote (BVLOS). Local inspections are intended to identify issues on the spot, in addition to recording data for further analysis. These operations are mostly flown in manual mode (pilot in control at all times, HI low ∈[1,3]) and some automation might exist in the sensors used to acquire data or flight control. The environment and mission carry a degree of complexity mainly due to the fact that the unmanned aircraft will be flying close to a structure that might be functioning (power pole, tower, bridge, factory chimney, etc.) leading to low-medium EC and MC ∈[1,6]. A remote inspection involves mainly data acquisition in large and remote areas. (note: it is unclear the benefits of UAS for inspection in urban areas over current methods, due to the strict regulatory frameworks currently in place). EC and MC can be relatively high (∈[7,10]) due to the lack of pilot visual contact with the unmanned aircraft and the precision requirements on the guidance and navigation, path planning, sense-and-avoid, emergency procedures, etc. Therefore, in a remote modality we propose the following configuration: high level tasks such as 1–11, 18 and 21, low level tasks common to most UAS such as 13–17 and mission safety tasks such as 20 (Table 1). In a local modality, we propose the following configuration, energy management (4), storage management (9), low level tasks such as 13–17 and safety tasks such as obstacle avoidance (20) (Table 1).
- *Precision agriculture* applications typically have strict requirements on data accuracy, timing, overlap and resolution, amongst others. Furthermore, these requirements impose additional constraints on the flight pattern (navigation and guidance tasks) performed by the UAS. If we assume a typical farm with an extended area and relatively free airspace, then EC and MC can be both considered medium. However, HI will be high, in most cases, due to the precise flight patterns needed to acquire accurate data. There might be cases in which manual flight is permissible (from the data requirements point of view) which leads to low HI. We assume this application is conducted within VLOS and the main sensor for data collection is electro-optical, leading to the following proposed task configuration for precise and safe navigation such as 3–6, storage management (9), low level tasks such as 13–17 and camera specific tasks (18) (See Table 1).
- *Parcel delivery* involves the transport of packages, food or other goods. There have been trials demonstrating the feasibility of this application in rural and suburban areas (low-medium MC and EC), and in close proximity to the base [31]. However, extending this application to a wider area (wider population) using more UAS simultaneously will likely require more onboard automation and support systems than currently in place. Systems such as air traffic management, collision and obstacle avoidance, decision making, etc. will now be required, which in turn will lead to more onboard autonomy (HI high). In our case study (Section 5), we assume a more generalised application in which HI is high, EC and MC are medium-high. Therefore, we propose a similar configuration to the remote inspection task, except for the need to run a specific task to land the unmanned aircraft at the delivery address (see proposed approach [31]). Assuming an electro-optical sensor might be used to the detect the landing site, we propose the following tasks 1–11, 13–17, 20–21 and camera used for landing (18) (See Table 1).
- *Aerial photography* is a common VLOS application in which cameras (still or video) are used to capture the scene. This application is typically conducted by highly skilled pilots (low HI) flying the UAS close to the movie scene (filmmaking), landscapes or man-made structures. Some automation might exist in the onboard cameras or gimbal but not enough to require a highly autonomous UAS. EC and MC are also relatively low due to simplicity of the overall task. Some might argue that the right positioning of the unmanned aircraft is critical to capture the best media possible, which in most cases it is true, however currently this requirement is handled entirely by the pilot and feedback through the video link. In this case, we propose the following configuration energy (4) and storage management (9), assuming an autopilot is used for UAS stabilisation, camera positioning and augmented flying then tasks 13–15 and 17 will be present, if a gimbal is used to track objects or fix the scene view independent of aircraft pose, then task 18 will be present (See Table 1).
- *Drone racing* is a relatively novel application of UAS. It consists of small multi-rotors equipped with controller boards for high speed precise and agile flight, and cameras to enable first-person-view (FPV) piloting. Almost all multi-rotors used in drone races are flown manually using FPV (low HI). The environment consist of several obstacles designed for high difficulty leading to high EC. The mission is however to complete the course through several checkpoints at high speed in a minimum time, therefore we assume a relatively straight-forward mission (low MC). The proposed configuration tasks will include energy management (4) and low level tasks such as 13–17 to enable unassisted rate mode [90]. Additionally, we can assume FPV and video recording fall under the category of application specific camera tasks (18) (See Table 1).
- *Search and rescue* using UAS is intended to aid and support search efforts in many situations for a fraction of the cost in terms of risk and resources. This application is considered very similar to remote infrastructure inspection (tasks 1–11, 13–17, 20–21), except for the addition of a camera task (18) to identify and localise objects or humans in the camera field of view. Environment and mission are considered high due to the coordination required (high MC) with other rescue systems that might be flying simultaneously (high EC). Human independence requirement is also high due to BVLOS operation modality (See Table 1).

**Figure 5:**
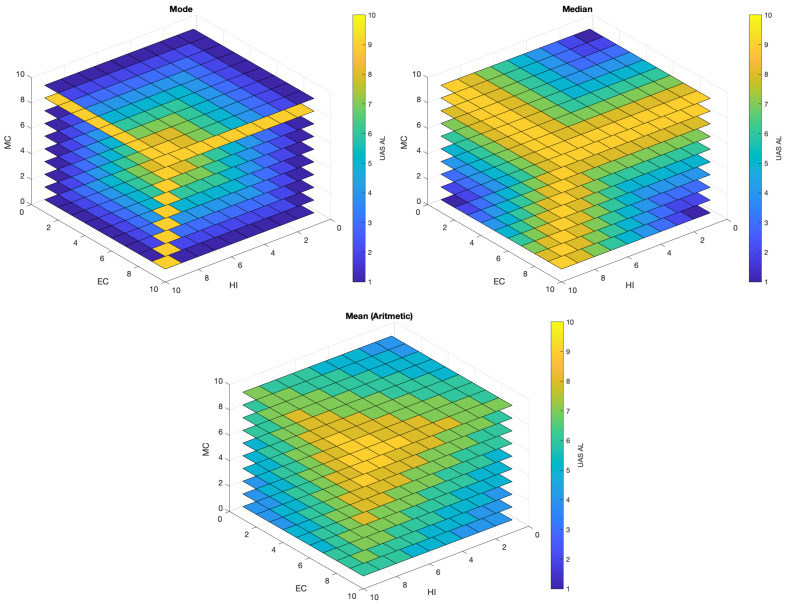
Estimation of the UAS AL using mode, median and mean (arithmetic). We note the arithmetic mean provides good balance between over/under estimation.

Based on the description provided for each application. We assign an integer value in the range [1,10] to each of the three aspects of the ALFUS model, HI, MC and EC, for each application (see Figure 4 and Table 2). Based on experience [26,34,76], we propose a number of tasks required to ensure feasibility of each application. We then provide three ways to estimate the UAS AL metric, mode, median and mean (arithmetic) of the three values for HI, MC and EC. Since there are multiple ways to find the central tendency for these values, we find the arithmetic mean (rounded to the nearest integer) provides the best balance between under/over estimation of UAS AL. In Figure 5, we show the estimation of these values using a scale 1 to 10. We can observe how the arithmetic mean can provide smoother transitions between levels and it is less biased towards HI. The UAS AL allows us to relate an application with a required autonomy level and the number of tasks $N_{t}$ to achieve it. We have observed a level of proportionality between the UAS AL and the number of tasks (or functions) a UAS should implement. The UAS AL is not a prescribed metric but rather a suggestion on how to map every aspect of the ALFUS model to a numerical scale that can be readily used during the hardware and software design process. We acknowledge this metric may be exposed to subjective bias due to the assumptions made by engineers and technologist when designing the subsystems to address a particular task. In any case, having a relationship between autonomy level and number of onboard concurrent tasks ($UAS\;AL\propto N_{t}$) allows us to draw a relationship between the autonomy level and the onboard computational resources needed to achieve it.

### Impact on Embedded Processing

Higher UAS AL means an increased demand for computational resources due to the large number of functions required to achieve high autonomy. Computational resources are provided by the onboard navigation processor (autopilot) and the mission board (embedded PC). The performance required by high UAS AL will go beyond the capabilities provided by current embedded PCs and cannot be addressed by simply adding more onboard computers due to SWaP constraints. Dedicated computing architectures for a single application are difficult to design, manufacture and are not cost effective. Some alternatives have been proposed using standard computers architectures. For example, Bonasso [91] pioneered the three tiered (3T) autonomous intelligent control architecture that has been the point of reference for various robotics implementations. Although this architecture has been designed and tested on ground robots only, the 3T paradigm can offer benefits to UAS. However, the design philosophy must account for the complex dynamics and kinematics of most unmanned aircraft and the environment in which they operates. For instance, unmanned aircraft operate in a highly dynamic and shared environment with greater velocity, unmanned aircraft have more degrees of freedom than ground robots and small to medium unmanned aircraft are also highly constrained in terms of payload and safety. Some dedicated solutions have been proposed recently to improve autonomy and safety by means of more embedded computing capacities. These works mainly address the implementation of a specific safety task, which is the first mandatory step on the path to autonomy. For instance, in Barry and Tedrake [48] a collision avoidance system based on stereo vision was demonstrated. Two implementations were tested using an Odroïd board based on a quad-core Cortex A9 Exynos SoC and a small lightweight FPGA. Both running at 120 fps on 320 × 240 image resolutions and tested onboard at speeds over 13 m/s. In Lai et al. [36] a highly accurate sense-and-avoid solution is detailed and demonstrated and implemented on a GPU board. Finally, an obstacle detection task running in all directions is described in Gohl et al. [92], the implementation is based on 4 Xilinx Zinq (hybrid CPUs/FPGA) and can process at 80 fps. It can be seen from previous examples the diversity of processing architectures ranging from CPUs, GPUs to FPGAs. It also highlights the tendency to use parallel processing architectures such as GPUs and FPGAs. Similar to the analysis made to define the UAS AL as a metric to convey a degree of autonomy for a given tasks, we are now interested to link autonomy with computational and hardware resources for applications that require a given autonomy level. In Section 5, we analyse the resource requirements for a representative case study namely Parcel Delivery, to evaluate the embedded processing requirements for this task. We expect to draw a relationship between the type of computing requirements and architectural model we can anticipate in future applications that need to achieve a level of autonomy.

## 5. Case Study: Parcel Delivery

### 5.1. Parcel Delivery

This application is one that has received considerable attention by society. We consider parcel delivery as one of the key BVLOS application in industry. In this section, we present a case study to highlight the requirements for a fully autonomous UAS for parcel delivery. We consider autonomy a capability that goes beyond waypoint navigation. As a first step, we consider a UAS with two onboard computers. The first, a type of embedded computer (autopilot) which handles most of the flight-critical related tasks. We assume the autopilot has an architecture similar to the one presented in Table 3, which are the most common architectures used in recent years. The second, a mission board (sometimes referred as companion/payload computer) that handles non-flight critical tasks (most high level tasks). This computer has a small form factor typical of industrial embedded PCs such as PC104, mini-itx, Atom^®^or Edison^®^ boards [36,93,94]. In Table 4, we extend on our analysis presented in Section 4 (Table 2) by describing the considerations and assumptions for each of the onboard tasks suggested for a parcel deliver drone.

From Table 4, we can observe that a number of tasks that are executed on the autopilot (7) and on the mission board (13), respectively. Intuitively, the autopilot deals with a smaller number of tasks compared with the mission board. In practice, most autopilots listed in Table 3 can handle this amount of computational requirements. However, the mission board will be running a number of tasks ranging from image processing and path planning to online estimators and decision making. We can already observe, although qualitatively, that unmanned aircraft with high UAS AL will require significant computational capability either in the form of customized hardware or commercial-off-the-shelf computers.

### 5.2. Assessing Task Computational Resources Requirements

Future UAS applications such as parcel delivery will require computing resources with different processing capabilities as highlighted in Table 4. Using the set of metrics we provide in Table 5, an indication of the complexity and resources required by some of tasks on an unmanned aircraft conducting a mission such as parcel delivery can be gained. Due to the number of possible implementations for a given task, it can quickly become impractical to provide a comprehensive list, however the implementations presented in Table 5 represent good indication of the computational loads expected for this application.

With regards to Table 5, we first consider the type of parallelism (Col. 2) using a couple of metrics that have a strong impact on the efficiency of the target execution platform. The type of parallelism focuses the usual difference between instruction and data streams [97]. In practice we consider control (C) (e.g., task) and data (D) parallelisms where we indicate the granularity of processed data that can be scalars (S) or vectors (V). We also mention the dominant hardware-independent parallelism model (Col. 4) by means of standard skeletons [98] at data (map, fork) or task (sequential, farm, pipe) levels. The resolution type (divide and conquer, branch and bound) is introduced as a type of “Dwarf” (Col. 5) according to the Berkeley classification [99], which is based on 13 typical families (called “Dwarves”) of benchmarks that aim at extracting processing and memory accesses patterns. Another indicator (Col. 3) is the main performance limit in terms of memory bandwidth or latency, number of processing elements, or the use of parallelism specified by designers. Beyond the distinction between scalar and vector, another important metric for the selection of the execution platform is the data format and so the type of computing that may or may not take advantage of FPU units to get the required accuracy (Col. 6).

An exact study of computation load in terms of operations would require the complete specification of the different tasks with a common high-level language to compute accurate parallelism metrics as described in [100]. This is hardly possible in practice because, firstly performances are strongly context-dependent, secondly most of application specifications are implemented using different programming languages in most cases not freely available, and finally new release versions with different implementation styles are regularly published. Our objective was not to measure the exact computation load which is fluctuating by definition but to provide an estimation within a range. We have considered and studied a selection of influential papers and contexts for each task to extract best and worst cases in terms of computing load. The context and system parameters (resolution, data rate, speed) are based on the parcel delivery case study. Finally, we obtain a coarse estimation of the computation load given as a range [min, max] OPS (Col. 7), which represents a typical MAC-type or FPU operation. This metric can be used to compare the complexity of functions required by mission, application, navigation, flight, safety, etc. Finally, based on the previous criteria, a designer can evaluate the matching degree of computation load with typical architecture models such as single or multi-CPU, FPGA or GPU. Multiple choices are possible when different tasks compete in terms of requirements. We have previously suggested a number of tasks that will increase onboard autonomy making the parcel delivery application more robust and dependable. Some of these tasks and possible extensions are now assessed in Table 5 using a number of metrics to attempt to provide an indication of the computational load of the overall application. We provide insights on this table and draw some perspectives about the target hardware architecture in Section 6.

## 6. Addressing the Computational Gap for High Levels of Autonomy

Table 5 allows us to draw some conclusions about the type of computing resources that would be necessary to execute a number of tasks in order to reach a given autonomy level.

### 6.1. Embedded Computing Requirements

#### 6.1.1. Computation Load

This parameter can exceed 110 giga floating-point operations per second (GFLOPs) when several applications run simultaneously. Currently, this type of performance is not accessible on embedded devices due to SWaP constraints. Recent peak performances on state-of-the-art GPU, CPU and FPGA seems promising reaching TFLOPS for basic operations [112]; however, these scores are far from those when real-world applications are considered. One of the main reasons is the memory wall, which prevents the full exploitation of the theoretical parallelism. Some dedicated architectures such as FPGA can meet performance expectations for some specific applications. For instance, a generic solution for deep-learning is proposed in [109] reaching 84 GFLOPs with the large and costly Virtex 7 device. In [113], the authors report impressive peak performances of 636 GFLOPs using an implementation of the Caffeine Deep Learning with the same device. They also show performance and energy gains over a 12-core Xeon server of 7.3× and 43.5×, respectively. Whilst performance is impressive, FPGAs in these cases run a single application at the time. Enabling FPGAs for on-the-fly reconfiguration in order to run several applications would be a highly desirable feature.

Meeting performance expectations with current multi-CPU, GPU or FPGA is difficult mainly because application’s parameters such as the computation pattern (i.e., Dwarf), floating-point computations, flexibility and other aspects described in [112], will vary significantly among applications.

#### 6.1.2. Heterogeneity

Based on the applications in Table 5, we can observe the different processing patterns and parallelism skeletons. However, there is one dominant class which is related to the “Dense Linear Algebra” Dwarf. It is indeed present in most computer vision applications that are based on MAP and PIPE parallelism skeletons. It is worth noting that Deep Learning-based applications also belong to this class. This class requires memory bandwidth and can benefit from data parallelism to achieve theoretical performances. Optimization and Decision making applications constitute another significant class of applications that rely on “Graph Traversal”, “Graphical Models” and “Monte-Carlo” types of Dwarves. They can also take advantage of FARM skeletons when distinct parts of the search space can be explored in parallel. Moreover, video processing and encryption are typical applications that can require high-speed processing rates, they belong to the “Combinatorial Logic” class and can take advantage of bit level operations and streaming data flow.

In summary, the target model of architecture must be considerably efficient to handle different types of processing schemes which means a hybrid model system on chip might be required. We also note that a enough flexibility is required to enable/disable the appropriate set of applications and configurations depending on mission phase and requirements. Beyond processing capabilities, the target embedded system must be flexible and optimized requiring advanced OS features and middleware to manage hardware and software reconfiguration as well as health and QoS functions.

#### 6.1.3. Memory Resources

Tasks such as computer vision, graph traversal and machine learning [114] are intrinsically memory-intensive. It results in highly critical on-chip storage as well as bandwidth requirements to access off-chip memories. Moreover, real-time computer vision means high bandwidth and low memory latency when reaction time matters (visual control, sensor fusion, fast detection). Three challenges are currently being addressed in this area, the memory wall problem, the on-chip/on-board memory static power consumption and overall memory capacity limits.

#### 6.1.4. Power Consumption

Depending on the type of UAS, the available power for embedded systems, sensors and communication links, may vary from few units (e.g., small hexacopter running on battery) to hundreds of Watts in large UAS. As a representative upper bound case, Boreal [115] is a civilian 4.2 m fixed-wing UAS with gasoline propeller, a payload of 5 kg and a power generator that can continuously deliver 100 W over 8 h. Based on the Top500 list [116], an efficiency value of 1 GFLOPS/W can be considered as a worst case scenario. An efficiency of 10 GFLOPS/W should be the target. This target is not possible with current CPU devices, but it can be achieved by FPGAs with dedicated architectures [117]. However, this optimization is nevertheless possible at the cost of re-designing the architecture every time the applications change.

### 6.2. Architecture Requirements and Proposal

#### 6.2.1. Architecture Model

As mentioned earlier, none of the processing architecture models (CPU, GPU, FPGA, dedicated HPC accelerators) can by themselves meet the expected performances required when a set of applications are running simultaneously. For instance, FPGAs can perform extremely well for applications that do not require floating point units (some types of computer vision) or computational logic applications such as encryption, providing the best power efficiency and I/O bandwidth. When floating point units are required, or efficiency in fixed point application is needed, then GPUs are well suited [112]. This is a typical case in the higher stages of computer vision processing after pixel level pre-processing, in some estimation problems, dense linear algebra methods and some deep learning applications. The computing capabilities and efficiency of embedded GPUs are still advancing and should be considered in any hybrid SoC. Finally, multi-core architectures are suitable when task/application management is required. Task activation/de-activation or re-configuration depending on the mission context is a process generally well managed by the operative system which traditionally uses the CPU for this purpose. Multi-core architectures are also the best for controlling sensors, managing communications, energy and handle complex HPC FARM-type applications such as multi-objective mission planning.

Data access or bandwidth is another key parameter that can increase when multiple cameras (multispectral and/or high definition) or additional sensors such as Lidars are considered. This implies that a possible computing architecture should have numerous and large on-chip memories requiring multiple controller ports to access memory. A Network-on-a-Chip (NoC) is an attractive solution that offers the expected bandwidth and allows multiple concurrent data transfers between distributed processing or storage resources. NoCs can handle dataflows that use both packet and circuit communications protocols. They are also well suited to handle mixed criticality flows [118] jointly with OS (criticality of tasks), for example safety tasks cannot be superseded or slowed down by other tasks. As previously suggested, multiple processing architecture models should co-exist within an embedded HPC system in order to meet expected processing demands of multiple applications running simultaneously. However, factors such as memory models (shared, dataflow), energy consumption, synchronisation, race conditions and data dependencies are difficult to trade-off in a multi-architecture processing system. Furthermore, the nature of parallelism, e.g., whether a program can be parallelizable or not (Amdhal’s law), will define the efficiency in a multi-processor model. As an alternative, we propose that a CPU-FPGAs architecture would overcome some of these limitations by being able to be dynamically reconfigured (FPGA dynamic reconfiguration). FPGA Dynamic Partial Reconfiguration (DPR) allows to reduce the FPGA size (large FPGAs are costly) and to maximize its use. DPR can be seen as a tile-based FPGA with multiple configuration ports that work like many embedded FPGAs in a single chip. A tile-based FPGA architecture also allows power gating when a resource is unused. It is also worth noting that available parallelism will be efficiently exploited is this approach if combined with a dedicated NoC that provide performance and flexibility comparable to NoC designed for multicore architectures [119] and not a light NoC [120] optimized but degraded to fit with FPGA resource. In terms of applications, FPGA still remains complex to program. The application complexity (design programming language) issue can be solved with an upgradeable library of IP cores designed for a set of standard functions/routines that will be mapped to the tiles on demand. To conclude, autonomy requires embedded intelligence. This means that complexity will grow over time including more sensors, data and more processing. In the UAS case, it means that SWaP will likely lead to FPGAs with DPR capabilities. The objective is then to design an architecture and programming methodology that makes this evolution possible and efficient.

#### 6.2.2. Towards Hybrid Reconfigurable Systems

**Figure 6:**
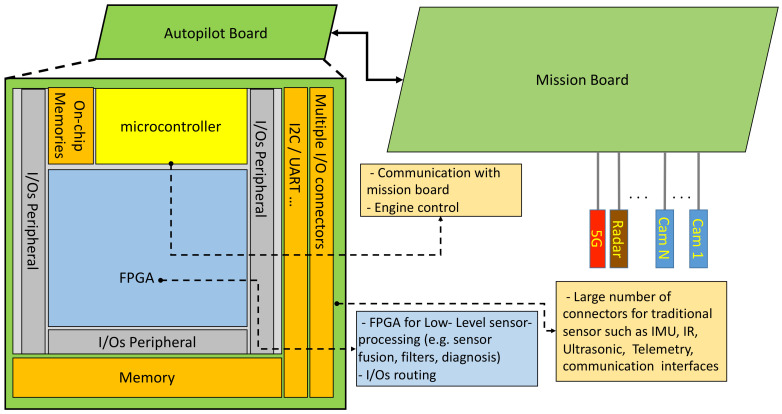
Architecture model for a reconfigurable autopilot board. Interaction and overview of the mission board is detailed in Figure 7.

**Figure 7:**
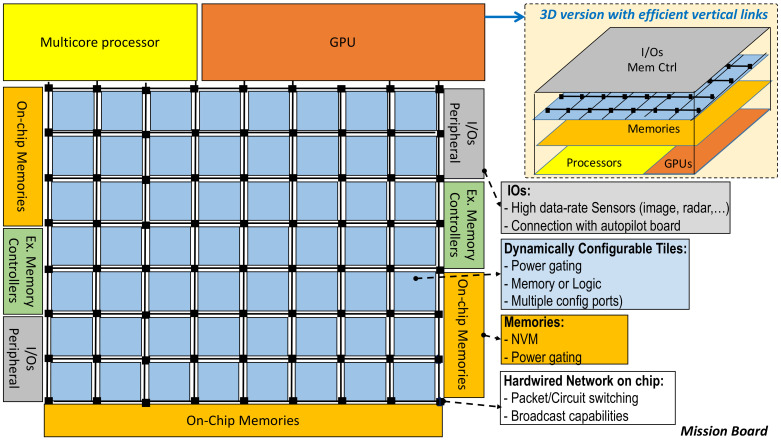
Model of reconfigurable hardware architecture for the mission board. Note the hybrid approach for the mission board, CPU, GPU plus reconfigurable FPGA.

The common approach in unmanned aircraft is to separate autopilot and mission boards. The autopilot is the reptilian brain of the aircraft, it is charge of critical basic tasks such as engine control, flight control and low-level signal processing and sensor fusion. The mission board is in charge of higher level and less critical tasks. Figure 6 visually depicts this approach. The autopilot can be one of the standard solutions given in Table 3. A mission board is based on an embedded multicore device that also includes a GPU co-processor (e.g., Odroid-based Exynos5 chip). These heterogeneous computers by themselves unfortunately do not meet the performance requirements of embedded computing for autonomy (Table 5). The solution will not be offered from this generation of such devices because of the energy efficiency and memory walls. An alternative approach will be to enhance this heterogeneous computers with reconfigurable hardware. This is the case of our proposed design for the mission board (see Figure 7).

A promising option would be to include an FPGA on the autopilot board as proposed by OcPoC [121]. Here the objective is not HPC, but to mainly take advantage of the FPGA flexibility to configure I/O connections according to a choice of sensors. Moreover, the FPGA also offers a solution to efficiently and locally implement computations related to basic and low data-rate sensors such as IMU, infrared, ultrasonic or optical flow. The Mission board is in charge of high-level tasks and intensive embedded computing. Based on our analysis we come to the conclusion that a hybrid and reconfigurable architecture with an efficient communication network is a promising architecture model. Considering the dynamic context of UAS missions, such a model can offer expected energy efficiency and performances to run many tasks (e.g., image processing, machine learning, graph traversal, etc.) that can fully benefit from FPGAs. Thus, each task can be dynamically configured in the reconfigurable hardware area of the FPGA according the mission requirements. Figure 7 presents an overview of this architecture model.

Heterogeneous SoC including GPU, multi-CPU and FPGA are already emerging. For instance, the Xilinx Zynq UltraScale+ EV is an example of such architectures designed for markets such as automotive (Advanced Driver Assistance Systems (ADAS)). However, some work still needs to be done in order to include NoC, more on-chip memory and a tile-based FPGA architecture that allows fast and concurrent reconfiguration as well as power gating.

Going in that direction, Xilinx has released in 2020 the heterogeneous Versal architecture [122]. This architecture actually implements a tile-based approach with an NoC to provide high-bandwidth and fast link to memories. This technology is dedicated to AI and 5G applications and it is implemented alongside conventional sub-systems such as CPU and configurable logic. A unified programming model is required for such complex heterogeneous system, in that domain OpenCL [123] is a promising initiative that paves the way to such a global approach. As an example of this proposed new architecture, we have started the development and test of dynamically reconfigurable hardware. We use ROS and interface it with the FPGA to take advantage of partially and dynamically reconfigurable hardware [124]. Such a complex system can only be developed by combining efforts and contributions from the research community. We believe the open-source community could be key in future development of this technology for general robotics applications.

### 6.3. Impact on UAS Design Methodology and Opportunities

New embedded computing architectures will have an impact in the overall UAS design. Traditionally, UAS design follows a Systems Engineering approach in which requirements and constraints are mapped into the avionics design [125]. For instance, Sandraey [126] lists fifty steps in their UAS design process which performs a hierarchical grouping of aircraft subsystems (wing, tail, engine, landing gear, structural configuration, autopilot) which are themselves decomposed into sub-subsystems and subject to optimization each. Interestingly, this approach rarely considers the payload and/or embedded computing system configuration and design in the process, as if flying was the only purpose of the mission. A similar approach was proposed by Schumann et al. [127] by associating a scenario-based statistical simulation to a quantified hierarchical description of the system to evaluate a global cost vs. interest of a UAS configuration.

More recently, Integrated Modular Avionics (IMA) design philosophy has been used in unmanned aircraft. This approach has a broader view on the system and takes into account not only aerodynamics and flight control but the embedded system and payload configuration. This approach has a long history in aviation [128]. This design approach favours the use of COTS hardware, follows software engineering practices such as code reusability, portability and modularity, and integrates safety, security and partitioning standards as well. All these design principles are now being adopted in a new generation of UAS (large, mini or micro).

Based on the observed design trend, we expect that IMA design philosophy for unmanned aircraft will continue to grow by using more COTS available systems. The emergence of more readily available software binaries for CPUs/GPUs or bitstreams for FPGAs will facilitate this process.

#### Towards Service Oriented Architectures (SOA) for Flexible and Adaptive Unmanned Aircraft Embedded Systems

The heterogeneity of missions envisaged for the use of UAS, some which are mentioned in Section 2, means that system modularity and flexibility must be a major design criteria. In this context, an SOA model for the onboard embedded system offers major benefits as a design philosophy. The definition of SOA has its origins in web services and applications. When extended to unmanned aircraft real-time onboard sub-systems, this model, as discussed in [129], raises several critical questions relating strict compliance with real-time and safety constraints.

The design philosophy depicted in Figure 6 and Figure 7 is based on a layered architecture model for interconnection of aircraft subsystems. This model aims at providing the same level of modularity and flexibility of IMA systems [130]. It also relies on the separation between the flight control system and the mission processor unit that controls a Mission-Oriented Sensor Array (MOSA). The physical separation of boards in charge of *flight control* and *mission/application* is motivated by safety and mission-flexibility requirements. For instance, including all tasks/applications (excepts for those that are low level such as flight control and stabilization) on a single but highly configurable SoC such as [122] would meet design and performance requirements. However, our proposal, which shares some features with [122], extends this philosophy to the unmanned aircraft autopilot and mission boards.

Such an architecture will benefit from off-the-shelf SW libraries and HW IPs, as well as from a single common high-level specification that can be compiled for three different targets such as FPGA, GPU and multicores. OpenCL has recently emerged as a promising unique specification which is already available on Xilinx and Altera-Intel tools. However, currently it still requires a high level of hardware expertise to reach expected performances [123]. Traditionally, the development of tools and libraries is driven by mass and high-growth markets. Luckily, the UAS field is one of such markets. Significant progress is for instance already visible for application domains related to UAS such as computer vision and machine learning.

Flexibility, safety, robustness, power consumption are all attributes that will shape the computing architecture design, however we believe energy efficiency and performance will drive the design and adoption of reconfigurable hardware. Therefore, efforts should be place in the safe control of the online HW/SW reconfiguration process (reconfiguration controller). A promising solution is to rely on autonomic computing techniques and formal methods to warranty the safe behaviour of the reconfiguration controller. A preliminary work on the reconfiguration controller using generic FPGA hardware is presented in [131,132]. In this paper, we use these concepts but focus a the hardware proposal that could be used by such reconfiguration controller. In [131], we present *Discrete Controller Synthesis* techniques to automatically generate a correct-by-construction automata that controls the loading of FPGA bitstreams at runtime. It is extended to the case of UAS in [132]. This is an important advance that will benefit greatly the creation of highly autonomous aircraft by making them more capable in terms of computing capabilities.

## 7. Conclusions

UAS are expected to provide a large set of valuable services in the near future, but the emergence of such services is conditional on autonomy levels since many of these services are likely to be beyond line-of-sight operations. These type of operations will require strict adherence to safety and regulation standards (not discussed in this paper).

This work has presented an analysis of the type of tasks that will be required to reach a desired level of autonomy, and the implications on the computing requirements to reach that level. We have provided insights on how these autonomy levels could be quantitatively mapped. This metric is not intended to be exhaustive, this is beyond the scope of this paper. However, by assigning quantitative values to each axis in Figure 1, an autonomy level required for a mission could be indicated. This autonomy level has implication on the computing resources available onboard. Using a case study, our study has relied on the analysis of state-of-the art typical applications that we consider as representative of tasks of the five identified categories namely: flight control, navigation/guidance, application, mission and safety. Based on our analysis and considering SWaP constraints, we come to the conclusion that a heterogeneous architecture with reconfigurable hardware would be highly applicable. This is even more the case for small-size vehicles.

We believe that UAS designers must now consider the embedded system as a core piece of the system. Similar to the emergence of FPGA-based architecture in data centres, the use of reconfigurable computing is a solution to the required performance with optimal energy efficiency. Such application domains should strongly favour the development of more efficient heterogeneous architectures and programming tools. 

## Figures and Tables

**Table 1:** Most common tasks executed in UAS.

Number	Task-$N_{t}$
1	Sense and avoid
2	Emergency procedures
3	Fault detection and identification
4	Energy management
5	Path planning and trajectory tracking
6	Waypoint navigation
7	Decision making
8	Computational resources management
9	Storage management
10	Communication management
11	Airspace management
12	Vision-based navigation
13	State estimation and stabilisation control loops
14	Actuator control
15	Low level sensor readings
16	Application specific sensor readings
17	Telemetry communications
18	Application specific camera tasks
19	Aerial sampling
20	Obstacle avoidance and terrain following
21	Onboard health management

**Table 2:** Relationship between type of application and number of suggested onboard tasks. In this table, Infrastructure Inspection is divided into remote (R) and local (L). UAS autonomy level (AL) shown is the mode, median, mean (arithmetic), respectively (see Figure 5).

Application	VLOS (Tasks in Table 1)	BVLOS (Tasks in Table 1)	ALFUS Complexity	UAS AL
Infrastructure Inspection (R)		$N_{1-11}$,$N_{13-17}$,$N_{20-21}$	HI = 8, MC = 9, EC = 9	9, 9, 9
Precision Agriculture	$N_{3-6}$, $N_{9}$, $N_{13-18}$		HI = 8, MC = 6, EC = 5	5, 6, 6
Infrastructure Inspection (L)	$N_{4}$, $N_{9}$, $N_{13-17}$, $N_{20}$		HI = 2, MC = 3, EC = 6	2, 3, 4
Parcel Delivery		$N_{1-11}$, $N_{13-18}$, $N_{20-21}$	HI = 9, MC = 8, EC = 6	6, 8, 8
Aerial Photography	$N_{4}$, $N_{9}$, $N_{13-15}$, $N_{17-18}$		HI = 2, MC = 2, EC = 2	2, 2, 2
Drone Racing	$N_{4}$, $N_{13-18}$		HI = 2, MC = 2, EC = 5	2, 2, 3
Search and Rescue		$N_{1-11}$, $N_{13-18}$, $N_{20-21}$	HI = 8, MC = 7, EC = 8	8, 8, 8

**Table 3:** Common off-the-shelf autopilot for unmanned aircraft under 20 kg.

Autopilot	Embedded System
MicroPilot ${}^{1}$	150 mips RISC processor
Pixhawk^®^ ${}^{2}$	32-bit ARM Cortex-M7 core with FPU and DSP, 216 MHz. 512 KB RAM. 2 MB Flash
APM 2.6 ${}^{3}$	Atmel’s ATMEGA2560: 8-bit AVR RISC 256 KB ISP flash memory
OpenPilot CC3D ${}^{4}$	STM32 32-bit microcontroller/90 MIPs–128 KB Flash/20KB RAM
F3 Flight Controller ${}^{5}$	32-bit ARM Cortex-M4 core/72 MHz–256 KB/2 MB Flash
OcPoC ${}^{6}$	Xilinx Zynq (ARM dual-core Cortex A9/1 GHz–85 K S7 Xilinx FPGA)

${}^{1}$http://www.micropilot.com/products-mp2028-autopilots.htm (accessed on 4 February 2021); ${}^{2}$https://docs.px4.io/v1.9.0/en/flight_controller/pixhawk4.html (accessed on 4 February 2021); ${}^{3}$http://ardupilot.org (accessed on 4 February 2021); ${}^{4}$http://opwiki.readthedocs.io/en/latest/user_manual/cc3d/cc3d.html (accessed on 4 February 2021); ${}^{5}$http://seriouslypro.com/spracingf3 (accessed on 4 February 2021); ${}^{6}$ https://aerotenna.com/ocpoc-xilinx-zynq-supports-px4-autopilot (accessed on 4 February 2021).

**Table 4:** Assumptions and considerations for each onboard task in parcel delivery UAS.

Task	Assumptions and Considerations
**Flight control**	
State Estimation and Stabilization Control Loops	Executed on the autopilot. This task is based on a type of Kaman filter. Stabilization is usually based on linear controllers such as PID.
Actuator Control	Executed on the autopilot. Consists of dedicated hardware that translate commands values into PWM (typically) signals and then sent them to the motors.
Low Level Sensor Readings	Executed on the autopilot. Dedicated hardware executing analog to digital conversion, signal conditioning, sensor sync for internal use.
Telemetry Communications	Executed on the autopilot. Low level routines that packet telemetry data and send it through a serial interface. It also includes routines for error checking and heart-beat monitoring. Used by higher level tasks for decision making.
**Navigation and Guidance**	
Obstacle Avoidance + Terrain Following	Typically executed in the mission board. Involves the use of sensors such as Lidar and cameras to create a navigation capability for BVLOS operations [29].
Path Planning and Trajectory Tracking	Executed in the mission board. It is computationally expensive task, requiring dedicated hardware in most cases [58]. Here, we assume this task deals with the dynamic and kinematic constraints of the vehicle to generate optimal paths. In this context, this task will generate optimal trajectories that minimise time and avoid no-fly zones,
Waypoint Navigation	Executed in the autopilot. Computes heading and distance between a list of waypoints. It generates reference signals for the low level control, normally without dynamic and/or kinematic considerations. In this context, waypoint navigation will process pairs of waypoints to generate reference heading and velocity references that are proportional to the distance between waypoints.
**Application**	
Application Specific Sensor Reading	Typically executed in the mission board. Handles the connect/disconnect and reconfiguration of the sensor(s) being used. In this context, we assume an onboard camera is used for aided navigation (e.g., perform object detection and QR code recognition to land at the delivery address [95])
Application Specific Camera Tasks	Executed in the mission board. In this context, we assume a type of computer vision target detection and tracking is used by the UAS to land the drone at the destination [66]. We assume HD camera with a resolution of 720 p = 1280 × 720 pixels is used in this task.
**Mission**	
Onboard Health Management	Executed in the mission board. In this context, the system will monitor several onboard subsystems to detect anomalies that can impose risk to the mission. A type of probabilistic approach for decision making is common in this type of task.
Communication Management	Executed in the mission board. In this context, it will handle routines to re-establish the communication link in case of comms breakdown, data encryption on-demand and data integrity monitoring. Metrics computed by this task will define whether data compression should be adjusted, onboard data storage should be favoured over data transmission, etc.
Decision Making	Executed on the mission board. In this context, it will monitor other subsystems and communicate with other tasks to gather information that can be used to achieve a given mission goal(s). The overall goal here is to flight from the warehouse to the delivery address, several decisions have to be considered such as optimal flight path in consideration of battery level, no-fly zones and parcel weight. During flight, decisions need to be made in the event of unexpected malfunctions, changes in weather patterns and degradation of comms link.
Computational Resources Management	Executed on the mission board. In this context, it will evaluate the mission priorities at any given phase of the flight to allocate computational resources to tasks contributing to those priorities. e.g., allocate more CPU, GPU, FPGA resources to the detection and tracking task during the landing and delivery phase.
Energy and Storage Management	Executed on the mission board. In this context, this task will monitor the overall power consumption of the unmanned aircraft to enable/disable subsystems (tasks) based on peak power usage. It will also generate metrics to inform other tasks whether onboard storage could have priority over data transmission, compression or onboard processing.
**Safety**	
Sense and Avoid (SAA)	Executed in the mission board. In this context, this task will use a camera to detect other aircraft and generate avoidance commands to the low level control [36,69,96]. We assume HD camera with a 1280 × 720 pixels resolution.
Emergency Procedures	Executed on the autopilot and the mission board. Modern autopilots can provide capabilities such as return-to-land (RTL) or loiter that are configurable in case of telemetry loss, GPS signal loss or excessive wind. Advanced procedures usually require dedicated hardware and access to additional sensors (cameras, Lidars) to conduct more elaborated emergency routines [26]. In this context, this task will be executed in the companion board and the aim is to identify possible landing areas when an emergency landing is required. Additional, failsafe routines such RTL, Loiter, etc will be autopilot’s responsibility.
Fault Detection and Identification (FDI)	Executed on the autopilot and the mission board. Similar to emergency procedures, modern autopilots can provide some failsafe routines in case of excessive vibrations, uncalibrated sensors, excessive bias, or failure to read a given onboard navigation sensor such as accelerometers or gyros. A more elaborated approach could make use of estimators to detect actuators’ failure [73]. In this context, we assume a type of FDI is executed on the mission board to detect anomalies in actuators and sensors attached to this board. This task communicates with the health management task.
Airspace Management	Executed on the mission board. Use of a type of transponder either UHF (VHF) or cellular to communicate with a network in charge controlling UAS traffic [37]. Interaction with other traffic will have an impact in the path planning and waypoint navigation, therefore this task will communicate with other subsystems to help in the decision making during flight.

**Table 5:** UAS tasks for the case study: computation metrics are based on representative references and published metrics.

	Parallelism	Performance	Parallelism	DWARF	FPU	OPS	HW Target		
	Type	Limitation	Skeleton	Comput. Type	Needed	(Context)	MCU	FPGA	GPU
	Control	Mem. BW (MB)/Lat. (ML)	FARM/PIPE	Berkeley Classif.		Instr./s.			
**UAS Task**	Data (Scalar, Vect.)	Paral. (PL)/Comput. (CL)	SEQ/MAP	Attempt [99]					
**Flight**									
Trajectory Control	D(V)	CL	MAP	Dense Linear	Yes	[100 M–2 G]	×	×	×
(GPS/INS Filters) [39]				Algebra (DLA)					
**Navigation / Guidance**									
Egomotion Optical Flow [101]	D(V), P(V)	ML	MAP, PIPE	DLA	No	[1 G–10 G]	×	×	
ORB-SLAM	D(V), P(V)	MB, PL	MAP/PIPE	DLA ; Graph	No	[500 M–2 G]	×	×	
Monocular [102]			FARM	Traversal (GT)					
Obstacle Avoidance	C	ML	SEQ	Structured	Yes	[100 K–1 M]	×		
2D Lidar [29]				Grid					
Mission Plannning	C, D(V)	ML, CL	MAP	GT	No	[100–500 M]	×		
Multi-Objective [103]			FARM						
Path Planning BFS [104]	C, D(V)	ML, CL	MAP/FARM	GT	No	[100 M–1 G]	×	×	
**Safety**									
Vision-based	D(V), P(V)	ML, CL	MAP	DLA	No/Yes	[2 G–8 G]		×	×
Collision detection [36]			PIPE						
Visual-Sense and Avoid [96]	D(V), P(V)	ML, CL	MAP, PIPE	DLA	No	[3 G–10 G]		×	
Actuator Fault Detection [73]	D(V)	CL	MAP	DLA	Yes	[10 M–20 M]	×		
Emergency Landing CTL [26]	C	PL	FARM	DLA	Yes	[100 M–1 G]	×		
Landing Site Detection [105]	D(V), P(V)	CL	FARM / PIPE	DLA	No	[200 M–2 G]		×	
**Application**									
Object Tracking (DTL) [66]	D(V), P(V)	PL, MB, CL	FARM, PIPE	DLA	No	[5 G–10 G]	×	×	
QR Code [106]	D(V), P(V)	MB	MAP	Combin. Logic (CL)	No	[1 M–5 M]	×	×	
R-CNN People Tracking [107]	D(V), P(V)	MB, CL	MAP/PIPE	DLA	(Yes)	[2 G–10 G]		×	×
**Mission**									
Health Management [33]	D(S), P(S)	MB	PIPE	Graphical Model	(Yes)	[10 M–50 M]	×	×	×
Online POMDP Solver [108]	D(S), P(S)	CL	PIPE	Monte Carlo	Yes	[200 M–7 G]	×		×
Discovery DL [109]	D(V), P(V)	MB, CL	MAP / PIPE	DLA	(Yes)	[5–40 G]		×	×
RT Scheduling and	D(V)	PL	SEQ, FARM	Branch and Bound	No	[500 M–1 G]	×		
Resource Allocation [110]									
Video encryption [67]	D(V), P(V)	ML, MB	PIPE	CL	No	[0.75–10 G]		×	
Online Energy Management [111]	D(V)	PL	SEQ	Global Optimization	Yes	[0.5 M–3 M]	×		

## Data Availability

Not Applicable.
